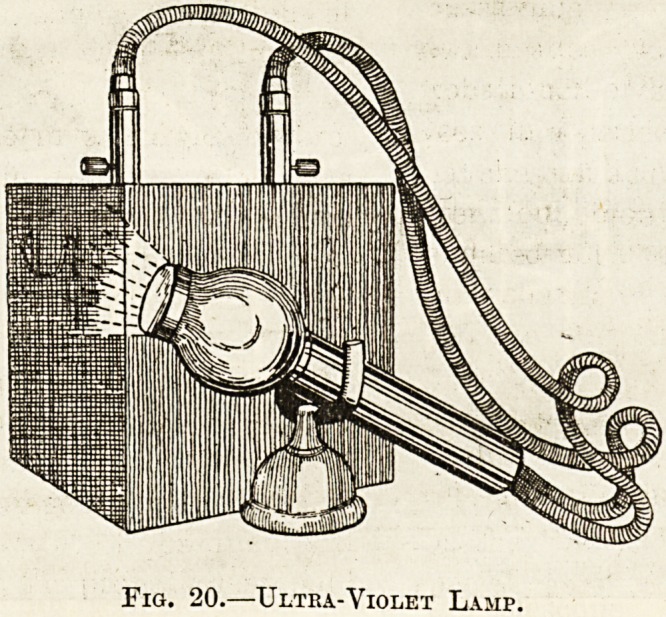# The Hospital. Nursing Section

**Published:** 1905-09-23

**Authors:** 


### The Hospital.

### Nursing Section.

Contributions for this Section of "The Hospital" should be addressed to the Editor, "The Hospital" Nursing Section, 28 & 29 Southampton Street, Strand, London, W.C.

No. 991.—Vol. XXXVIII. SATURDAY, SEPTEMBER 23, 1905.

### Notes on News from the Nursing World.

### AMATEUR NURSING OF A BROKEN THIGH.

It was announced last week that the Duchesse de Guise was thrown from her horse while she was out riding near Woodheaton in Worcestershire, and broke a thigh. The fracture, it was added, is simple and "it is not intended to engage a professional nurse, as this duty is being performed by the patient's mother, the Comtesse de Paris, and her sister, the Princess Louise." The accident occurred on Thursday, and on Monday it was intimated that an examination by the Röntgen rays showed that the fracture was rather more serious than at first supposed. The medical attendant, however, advised the Duc de Guise that in six weeks the Duchesse would be able to be about again. But in the circumstances she needs skilled surgical nursing. The fracture of a thigh is just one of those disasters which need constant care and attention, such as no one but a fully-trained nurse can give.

### A REAL GRIEVANCE AT HULL.

The Hull Guardians at their meeting last week were occupied for a long time in discussing a question which, in our opinion, should have been disposed of in five minutes. The nurses attached to the Poor-law Infirmary have been provided with a comfortable new home in Lansdowne Street through which, however, access can only be gained by passing through the lodge gates of the workhouse. The superintendent nurse wrote to the Chairman of the Staff Committee asking that they might be allowed to enter by the front door, a register being kept of every person entering or leaving the home; and the Staff Committee recommended that the request should be acceded to. But on the matter being brought before the Board a hostile resolution was moved, and supported by several guardians who professed themselves supremely concerned to maintain the authority of the workhouse master. The Governor, in pointing out that if the nurses' home had been built a little distance away from the workhouse they would naturally have used the front door, said that, if the nurses were not allowed to enter by the front door of the home, they would have to go through the lodge gates, and be signed on the register by an inmate. "That, he considered, would be somewhat degrading to the officials." Moreover, he thought that they should be made to feel that they were not prisoners. Other guardians spoke up bravely for the nurses, but the hostile resolution was carried by a majority of three; and unless it be rescinded at the next meeting, the boundary wall of the workhouse will, we understand, be so extended as to enclose the home and secure one entrance, through the lodge to the workhouse. We are greatly surprised that the Hull Guardians, who admit that they possess an excellent staff of nurses from the superintendent down to the probationers, did not unanimously and as a matter of course, assent to the reasonable proposal endorsed by their own committee. The refusal of the majority strikes us as an act of petty tyranny.

### THE TRAINING IN SMALL HOSPITALS.

In an interview which our Commissioner had with the matron of the West Cornwall Infirmary the interesting question of training in small hospitals was discussed. It will be seen from the report of the interview that the system of three years' training is in operation at the hospital in Penzance, which, like so many other institutions, has had to increase its accommodation, in fact to rebuild. The matron, however, with a candour which others might emulate, says that she is always careful to impress upon prospective probationers that the certificate they will get at the end of their term will not be equivalent in the eyes of the world to that obtained at a large hospital. But her point is that for those who do not wish for the rush and excitement which are characteristic of a large hospital, or who think it probable that they will afterwards go in either for a cottage hospital or for district or private work, the training given will stand them in good stead. Having so comparatively few patients at the West Cornwall Infirmary, it is possible, she affirms, to dispense with hard-and-fast rules and to study the wants, if not the fads, of those who are sick, in a way which is highly desirable in a private house, but is quite impossible in a large hospital. There is a good deal of force in this plea for the utility of small hospitals as training schools, and we see no reason why carefully-managed institutions of this class should not turn out nurses excellently equipped for private work.

### ENGLISH METHODS IN FRENCH HOSPITALS.

It is very pleasant to hear that the introduction by Dr. Lande of English nursing arrangements into the Tondu Hospital at Bordeaux has been already attended by the most satisfactory results. In twelve months, says Dr. A. Lutaud, of Paris, the hospital has been completely transformed, and visitors will now see there a number of young women cheerfully carrying out duties which hitherto it had been deemed impossible they could perform, while everything in the place breathes of moral and physical cleanliness. Dr. Lutaud hopes that the reforms thus successfully initiated will spread throughout the French hospitals, and he strongly advocates nursing as a career on the part of well-educated young women belonging to the middle classes in France.

### HOME NURSING UNDER THE LONDON COUNTY COUNCIL.

Evening classes under the London County Council have just been started for the winter session, and amongst other items "Home Nursing" is to have a place in the list. Female students over 16 years of age, at the cost of one shilling for the series, can join the course of lectures, delivered in some cases by a lady doctor. The subjects will include the home treatment of minor ailments, the care of infants and young children, the preparation of a sick-room, suitable diet, etc. The fee of one shilling will cover instruction on other subjects, the classes for which are held on different evenings to the home nursing lectures, and an optional examination will be held at the end of the session for awards, the Council's certificates, and medallions. It may be hoped that if this series proves successful there may be employment for a few trained nurses who have given up regular work.

### EXONERATION OF A SUPERINTENDENT NURSE.

At the last meeting of the Chichester Guardians Mr. Butler proposed that the superintendent nurse should be asked to send in her resignation on the ground that she had not used discretionary power in removing a woman from her own house without consulting her friends. The woman in question died a few hours after her admission to the workhouse, but the attempt of Mr. Butler to impute blame to the superintendent nurse was viewed with marked disfavour by the rest of the Guardians, none of whom rose to second his proposition. The chairman, replying to the attack upon her, said that she acted under the instructions of the medical officer and had merely done her duty. His practical reply to it was to move that the best thanks of the board be given to the superintendent nurse for the way in which she carried out her duties to the patients in the workhouse and outside. The resolution of thanks was carried, Mr. Butler alone dissenting, and there appears to be no justification whatever for his demand that she should be called upon to resign.

### LOYALTY TO THE DOCTOR.

The importance of loyalty to the doctor on the part of the nurse cannot be exaggerated, and there are seldom occasions when she has to choose between loyalty to him and duty to her patient. But Dr. Haynes of Los Angelos, in a recent address to the nurses of California Hospital, mentions an instance in which such a conflict arose. A medical man, he states, drove up to a house, tied up his horse without gloves upon his hand, and came to the bedside of an obstetrical case. The nurse—a California graduate—had in readiness three basins, with green soap and antiseptic solution. She said, "Doctor, here are the water and soap." He replied, "I will use those after I get through the case." The nurse rejoined, "Stop, doctor, you or I must cease in attendance upon this case, and the patient shall decide." The nurse then told the patient and her mother of the danger to the patient from being treated with unwashed hands, the result being that the doctor was asked to retire. Assuming the facts to be correct, we agree with Dr. Haynes that the nurse was right. But her experience, we imagine, was very unusual.

### SQUABBLING AT AUCKLAND WORKHOUSE.

As the result of an official inquiry by two inspectors of the Local Government Board into certain proceedings of the superintendent nurse at Auckland Workhouse including an altercation with the matron, the superintendent nurse has been censured by the Board for the use of improper language and has been pronounced guilty of an error of judgment in not sending for the medical officer in the case of a patient, and in failing to take measures to alleviate his distress pending the arrival of the medical officer. The Board found, however, that there were mitigating circumstances, that the master showed little self-restraint, that the superintendent nurse had been misled by the opinion previously expressed by the medical officer, and that the administration of the sick-wards had improved during her term of office. Her resignation was not therefore demanded, but both she and the master have been warned by the Local Government Board that in the event of further friction, the question of changes in the staff may have to be considered. The Board also suggested that the Guardians should themselves consider whether some of the nurses were not, by reason of inefficiency or of failure to understand the position and duty in relation to the superintendent nurse, an obstacle to the successful management of the sick-wards. The letter from Whitehall has been read to the superintendent nurse and the master, and the latter portion of it has been referred to the nursing committee. We think that great pains have been taken by the inspectors who made the inquiry to be fair to every one, and we hope that the practical outcome will be the cessation of squabbling.

### TYPHOID EPIDEMIC AT LIMERICK.

An epidemic of typhoid prevails in Limerick. The nursing of infectious diseases in this town is almost entirely in the hands of the sisters of St. John's Hospital, a picturesque building consisting of two distinct blocks for general and fever patients. The nuns themselves, who belong to the Order of the Little Company of Mary, are well trained, and are keenly interested in the modern developments of the art of nursing. They have regular courses of lectures by the visiting physicians and surgeons. Last week there were 34 cases of typhoid under treatment in the fever wards. When these are full, applicants for admission are sent on to the Union Infirmary. St. John's Hospital also supplies private nurses, and there is a convent belonging to the same Order which employs a certain number of nuns as district nurses.

### CONTINUOUS NIGHT DUTY.

The Faversham Guardians have decided to appoint two nurses for the Poor-law Infirmary, because the medical officer expressed the view that

"it would be difficult to get a nurse to devote herself entirely to night." Whether this be so or not, it would certainly be a most inconsiderate requirement. The regulations of a properly conducted institution would not permit a nurse to do night duty continuously for more than six months at the longest. As a rule three months is quite long enough, and even this period is often sufficient to produce anæmia and other signs of a lowered constitution. There should, of course, always be a nurse in attendance during the night, and there should also be one ready to take her place in the event of illness or accident.

### THE PENSION FUND AT BIRMINGHAM.

By the kind permission of the authorities, Mr. Louis H. M. Dick, secretary of the Royal National Pension Fund for Nurses, will give an address at the General Hospital, Birmingham, on Friday, the 29th inst., at 3 o'clock. All nurses interested in the subject are invited to attend.

### DEATH OF A NURSE FROM CHOLERA.

We regret to hear of the death of Miss Charlotte Andrews, who went to India last October as a nurse of the Up-Country Nursing Association. Miss Andrews was attached to Lady Ampthill's Nurses' Institute, and when the cholera broke out in Madras she offered, with other nurses of the Institute, to attend patients suffering from cholera. Early on the morning she commenced her duties at the Church of Scotland Mission House, she was herself attacked by the disease, and died on the afternoon of the same day. In reporting her death to the committee of the Up-Country Nursing Association Lady Ampthill says that she was a most excellent nurse and had done much good work in Madras.

### BRISTOL GENERAL HOSPITAL.

At the half-yearly meeting of the Governors of Bristol General Hospital the chairman mentioned the urgent need of more room for the nurses, and stated that when the isolation wards were swept away it was intended to extend the nurses' home, which he was told would cost about £5,000. The last addition to the nurses' home was made seven years ago, and consisted of 20 bedrooms, a sitting-room, and a bathroom.

### INSTRUCTIONS TO MANCHESTER MIDWIVES.

There is no excuse for Manchester midwives making blunders and getting censured by the Central Midwives Board. The Corporation, at the instance of the Midwives' Supervising Committee, has issued a little blue book in which is set forth very clearly all a midwife has to remember. The rules of the Board, the cleanliness of the person, the cleanliness of the dwelling, the midwife's bag and its contents, the management of the labour and of the lying-in period, the care of the mother and the care of the child, the register of cases, notification, and the precautions to be taken by midwives in cases of puerperal fever, are fully treated and for fear doubt should still exist in the mind of any Manchester midwife the last words of the pamphlet are "midwives may consult Dr. Merry Smith at the Public Health Office on any matter pertaining to the Act on Saturday between the hours of 9 and 11 a.m." Feeling that the handbook would be extremely useful to midwives in other cities and towns, we have arranged, with the consent of the Manchester Corporation, for its republication by the Scientific Press, 28 and 29 Southampton Street, Strand, W.C.

### RATS AT ASTON INFIRMARY.

It was announced at a meeting of the Aston Guardians on Tuesday that rats have invaded the Workhouse Infirmary, "to the terror of the nurses." We imagine that if, as it is stated, the rodents run over the beds of patients at night, the terror extends to them. The visitation is not due to the antiquity of the infirmary, which is quite a modern building, but we warn the guardians that if they want to keep the nurses they must, by some means, get rid of the rats. Long hours of duty, hard work, and want of appreciation might be endured, but there are few nurses who would not draw the line at rats.

### A NURSE'S WEDDING.

An interesting wedding took place at Tormsham Church, Torquay, last week, the bride being Miss Berry, a member of the staff of the Nightingale Nursing Institute in Torquay, and the bridegroom Mr. Arthur Reed, Liberal candidate for the Tiverton Division of Devon. The chaplain of the Devon and Exeter Hospital was one of the officiating clergy; the bride was given away by Miss Wilson, the matron of the Institute; the bridesmaids were nurses; and all wore the light blue uniform of the Institute. Several other nurses were present at the ceremony, which was followed by a reception at the villa where the Institute carries on its work. The staff gave Miss Berry a number of articles in silver.

### THE L.O.S. AND THE CORONER.

At a Coroner's inquest in Stepney on Saturday, respecting the death of an infant—reported in one of the daily papers—a person who attended the mother gave evidence. Being asked whether she was certificated, she said, "I am L.O.S." Thereupon the Coroner inquired the meaning of the letters. The witness first replied, "I don't know; my mother belonged to it"; but the question being repeated, said "I think it means Licentiate of the Society of Observation." The Coroner then asked, "had the witness got a nurse's certificate?" and the woman, who did not say that she possessed one, rejoined, "Well, you see, all the doctors come to me, I do the work and they get the money."

### THE FIRST PARCEL!

A quick response has been made to our appeal for contributions to our Christmas distribution of clothing. Nurse Pace, of Croyde Bay House, North Devon, sends us the first parcel this year. We hope, in a week or two, to be able to acknowledge further contributions.

### SHORT ITEMS.

The honorary secretary of the Newport and District Nursing Society in the Isle of Wight, informs us that the organisation is not affiliated to the Queen Victoria Jubilee Institute.—The annual conference of the National Union of Women Workers of Great Britain and Ireland will be held at Birmingham from October 23rd to October 27th.

### The Nursing Outlook.

> "From magnanimity, all fear above;
> From nobler recompense, above applause,
> Which owes to man's short outlook all its charm."

### "SLAP-DASH" IN NURSES' TRAINING.

At a time when the best system of training for the probationer is being thrashed out in detail it may be well to contrast the old system with that which prevails at present in one or more of the large hospitals. We do not suppose that anybody will canvass the statement that the pioneer trainer of nurses was the late Mrs. Wardroper, who for many years governed the Nightingale School with wisdom and success. We propose therefore first to give the details of a year's training under Mrs. Wardroper as recorded by one of the ablest nurses ever trained at this school.

It was the invariable rule at St. Thomas's in those days that everything should be done thoroughly and that nothing should be hurried. Thus, each probationer went for at least one month in each ward. In the more important wards a probationer would remain for two months' training under the sister's supervision. Every sister and every staff nurse knew she would have her probationers for certain for one or two months as the case might be, and all the work and organisation of the ward were planned to fit in with this system. A probationer then would spend two months in a medical ward, two months in a surgical ward, that is in male and female wards. Then she would have one month in a male, and one in a female ward alternately until she had been under training for eight months, then she spent a month in obstetric and a month in the ophthalmic wards. The remaining two months would be devoted to special cases such as ovarians, amputations etc., and finally if she was a "special" she had the advantage of one month on sister's duties in a surgical ward. Subsequently if she remained in the hospital, the probationer would have experience in charge, night, and sister's duties. No probationer in those days had to perform night duty during her first year. It will be noted that under Mrs. Wardroper a probationer had opportunities for seeing most branches of nursing under circumstances best calculated to enable her to profit by her experience.

Let us now contrast this with what is called the "in and out" training now adopted at some hospitals. A nurse is placed first in a male surgical ward for fourteen days, she is then moved into a second male surgical ward where she remains for three weeks. Next she enters a male medical ward for a month, then she is transferred to a women's surgical for fourteen days. After one day in a women's surgical ward (all this time she would be on day duty) she passes for night duty to a male medical for six weeks, then has four nights in another medical ward, and then for six weeks in the children's ward. After one day in a male medical ward she returns to day duty in the women's medical for six weeks. Her next experiences are, say, three weeks in a male surgical ward and then a week in the isolation block. After three weeks in a female medical she returns to night duty for six weeks in two male medical wards, finds herself for a few nights in obstetric, then she spends eight weeks on night duty divided up between three male surgical wards, involving three changes for the probationer to new conditions during that time. After a fortnight's day duty in a women's medical she may spend a week in the isolation block, then a fortnight divided between two male medical wards, once again a week in isolation, then back for a similar period to a male medical, and again for another week to isolation. Then at length she is left in a male surgical ward for nearly three months, after which she is transferred from day to night duty in the same ward for a further three months. Returning to day duty she spends a fortnight in obstetric, and completes her two years' probationership by serving for nearly three months in a women's medical ward.

Will anyone deny that under the "in and out" training the changes enumerated in the second course must prove most trying for any but a very level-headed woman? What, for instance, can a probationer of six months' training learn in one week in a perfectly new ward? Again, is it likely that she can acquire useful knowledge on night duty after four months especially if this service is rendered not in one, but in several wards, to which she is changed about at short intervals? Note too, that the only children's experience gained during the two years would be on day duty, whilst it is proverbial that children are very different at night to what they are in the day time.

Training of this latter kind, too, is not complete, for the "in and outer" appears to get very few special cases, too few for a complete training. The impression this system gives to the trained observer is, that under it the nurse must frequently be used to work the wards as needed, and that so the question of her adequate training is relegated to the second place. We have known many excellent nurses who have survived the "in and out" system, though they have necessarily been somewhat slap-dash in their methods, and we incline to the feeling that of the two systems, the old, or Mrs. Wardroper's plan, is infinitely to be preferred by practical people who make the interests of the probationers and the quality of the training their first consideration.

### Medical Electricity and Light Treatment.

By Kate Neale, Sister-in-Charge of the Actino-Therapeutic Department, Guy's Hospital.

### IX.—FINSEN LIGHT.

The Finsen treatment consists, in a word, of the application to the patient of a strong beam of light focussed on the skin. The light need not necessarily be derived from an electric lamp, for sunlight itself would answer as well and is infinitely cheaper. It possesses, however, the very great disadvantage of unreliability, and in England at any rate, its use is almost put out of court on this score alone. However, a brief account of the properties of sunlight as far as its use in treatment is concerned is necessary to help you understand the effect of the Finsen rays.

Sunlight is, as you know, white, and yet if you look through a glass prism at any object you see it edged with all the colours of the rainbow. In other words, the white sunlight reflected from the object becomes broken up in its passage through the prism into coloured light. But not only can this "decomposition," as it is called, of white light take place, but different coloured lights can be blended to produce white. We see then that what we are accustomed to call "white" light is really a mixture of many coloured lights, and this you can easily prove for yourself. Darken a room so that the light enters only through a hole in the shutter. You will be able to see a beam streaming across the room to the opposite wall, which it marks with a bright white spot. Now hold a prism in its path; the white spot disappears and in its place come the rainbow colours—from left to right on the wall, red, orange, yellow, green, blue, indigo and violet. This sequence of colours is spoken of as the Spectrum.

How is it then, if sunlight be white, that it can make some things look red, others green, and so on? A meadow looks green because all the rays of the white sunlight bathing it are absorbed except the green and these are reflected. Red, orange, yellow, blue, indigo and violet, all are taken in by the grass and retained and only the green is allowed to reach your eyes. You cut your finger and the red blood wells up—red because blood absorbs the colours from orange to violet, and permits only the red rays to be reflected.

Let us go a little further in our investigation of sunlight. We have seen how it consists of rays of seven colours, but there is even more in it than that. Put a basin of water out in the sun, and it will become warm. Sunlight then possesses **Heat Rays**. Sit out in the open under an August sky without any sunshade and your skin will become tanned and blistered. The sun then has **Chemical Rays** which can blister the skin. Light rays, heat rays, chemical rays are all present in sunlight. Cures by Finsen treatment are effected by the chemical rays.

It has been found that the heat rays are always associated with the red light rays—*i.e.* they occur at the red end of the spectrum. This relation of heat to red rays is unconsciously recognised in many incidents in every-day life. No one, for instance, likes to see a girl dressed in scarlet on a summer's day, yet in mid-winter nothing is more grateful to the eye. Similarly the chemical rays are restricted to the violet end of the spectrum, though, in addition, they extend further to the right where they become invisible, and are known as the **Ultra-violet Rays**.

Artificial light (electric, gas, candle, etc.) is made up of the same seven colours as sunlight, but the proportions of heat and chemical rays in them varies. Gas light, for instance, contains many red and yellow rays, and must therefore give out much heat but few chemical rays, and will be of no service for Finsen treatment. The ordinary incandescent electric light used in private houses is useless for the same reason, but the bluish light from electric arc-lamps such as you find in streets and large railway stations, is very rich in chemical and ultra-violet rays. The arc-light then is the one used in treatment.

### Apparatus Employed in Treatment.

There are three varieties of lamps you will work with. The original and most important is the Finsen lamp. A modification of this is the Finsen-Reyn lamp, while a third form is called the Lortet-Genoud, or French lamp. In addition to these the Leslie-Miller lamp produces ultra-violet rays alone.

I. **The Finsen Lamp** (see illustration).—The light is derived from two pieces of carbon, the pointed ends of which are set close together. On passing a strong current through them their ends grow white hot, and the space between them is filled with a dazzling light rich in chemical and violet rays. These carbons are surrounded by the metal cylinder seen in the figure suspended from the ceiling. The light is led down each of the telescopes that radiate from the carbons, and is finally focussed on the skin of the patient lying beneath.

Each telescope is temporarily closed at its lower end by a perforated metal cap, and contains a series of lenses, made of rock-crystal in preference to glass, as the latter interferes with the passage of ultra-violet rays. These crystal lenses are easily cracked, and have to be protected from the intense heat of the arc-light by filling both the upper and lower halves of the telescope tube with distilled water, and circulating round it a stream of cold water led to and from the tube by rubber piping. This arrangement serves not only to keep the lenses cool but also to absorb the heat rays before they can reach the patient, whom they would burn.

On emerging from the lower end of the telescope the rays have to pass through yet another lens, known as the **Pressure-Lens** (fig. 17). This consists of two pieces of rock-crystal set in a metal ring and separated from each other by an interval through which more cold water flows. Rising from the side of the metal ring are four handles. The lower of the two crystal lenses is either convex (*i.e.* bulging), concave (*i.e.* hollow, like a saucer), or flat. A choice of pressure-lenses can therefore be made according to the shape of the surface to undergo treatment. Thus, if you were treating the tip of the nose, a concave lens would fit much better to its curve than a convex, but the latter would be preferable for a receding surface, such as the side of the neck. Though the pressure-lens is used in part to keep the patient's skin from being burnt by the lamp rays its further function is to compress the skin and drive the blood out of it. From what we saw earlier in the chapter you will understand that since it is the violet rays which are of prime importance in treatment, it would be futile to try to send them through any tissue containing red blood which would absorb them and not let them through. The pressure-lens then is to be always pressed tightly and firmly against the skin to render it bloodless.

The light from the Finsen lamp is equivalent to thirty thousand candle-power, and is far too intense to be looked at by the naked eye. All nurses therefore must wear dark green glasses to protect their sight. It is most unwise to remove them during a treatment with the idea of seeing what the appearance of the skin is, for not only do you run great risk of headache with redness and inflammation of the eyes, but the aspect of the skin is so different with and without glasses that your judgment becomes confused.

Lastly, the cost of working a Finsen lamp is not small and you must so arrange your cases that all four telescopes are in use so long as the light is on.

FINSEN LAMP.

II. **Finsen-Reyn Lamp** (fig. 18).—This is a much smaller lamp than the Finsen, and only one patient at a time can be treated. Its candle-power is three thousand. It consists of an arc-lamp, and a short telescope fixed to a movable stand. The telescope contains rock-crystal lenses and is provided with a cold water circulation. This is of especial importance because the distance between the carbons and the first lens is small—a matter of inches—and there is great risk of the lens cracking. The pressure-lens is used with this lamp as with the Finsen.

III.—**French Lamp** (Lortet-Genoud). — This variety treats a larger area at one time than either of the preceding forms, but it is not so deep-reaching in its action and is therefore most frequently used for superficial affections of the skin. The light is again provided by the carbon points of an arc-lamp, but there is no telescope (fig. 19). In front of the carbons is a conical hollow metal shield, perforated in the centre by a window, in which are fixed two rock-crystal lenses. The space between the lenses is continuous with the interior of the shield, and through them both a circulation of cold water is maintained by two rubber pipes. The carbon points meet at an angle, and can be separated or approached by turning the handle A. The other handle B slides them nearer to the lenses.

FIG. 17.—PRESSURE-LENS.

No pressure-lens is required with the French lamp as the necessary pressure is obtained by bringing the front lens of the shield against the patient's skin. To allow of this the lamp is fixed on an adjustable stand.

IV.—**Ultra-Violet Lamp** (Leslie Miller).—This instrument, depicted in fig. 20, consists of a tubular handle, one end of which is closed by a rock-crystal

### MEDICAL ELECTRICITY AND LIGHT TREATMENT—*Continued.*

lens. Behind the lens are, instead of carbon points, two iron points, and these communicate at the far end of the handle with two wires leading to a box

FIG. 18.—FINSEN-REYN LAMP.

called a "condenser." Again no special pressure-lens is needed, the pressure of the crystal lens sufficing to render the part bloodless. The peculiarity of the instrument is that the sparks which pass between the iron points give off a light particularly rich in ultra-violet rays.

### Lupus.

The disease for the cure of which the Finsen treatment is so efficacious is that known as lupus, and a brief account of its nature will be of service. Lupus is a disease occurring especially in childhood or early adult life. It begins as a small nodule in the skin which, breaking down, leaves a raw surface. Around the edge of the ulcer thus formed fresh nodules appear and they in turn ulcerate, leading to an increase in the size of the patch. Thus the disease tends ever to spread, slowly eating away the skin as it progresses until, finally, large areas—a whole cheek or even half a face or more—may be destroyed. The cause of the condition is the tubercle bacillus (the same bacillus that invades the lung in phthisis), which, settling in the deeper parts of the skin, produces the above changes. Though the face is the commonest site of the complaint neither trunk nor limbs are exempt.

The chemical rays from the Finsen light possess

FIG. 19.—LORTET-GENOUD LAMP.

the power of destroying these bacilli and at the same time of setting up an inflammation ("**reaction**" as it is called), which finally leads to the

FIG. 20.—ULTRA-VIOLET LAMP.

healing of the skin. Because the disease spreads by the small nodules at its edges treatment is always applied to these nodules, and thus further extension is prevented.

### The Nurses' Clinic.

### THE CARE OF SPLINT AND EXTENSION CASES.

THERE are many points to be noted in the nursing of patients who have one or more of their limbs encased in splints, as the neglect of certain precautions in these cases may lead to unlooked-for if not serious results. First, with regard to the padding of splints, nothing better can be devised for this purpose than well-teased-out tow, covered with soft old linen. Splints should be evenly padded, and sufficient padding must be used. It is far better to err on the side of too much than too little. I have seen a large blister on the leg of a child as the result of insufficient padding of a splint; fortunately in the case in question the complaint of pain was heeded in time, the splint temporarily removed, and the blister dressed with suitable ointment, thus avoiding a more or less painful ulcer. Nurses will do well to remember that pink jaconet should never come in contact with the skin, especially the delicate skin of a child; when it is necessary to cover a splint with jaconet, either place wool between the jaconet and the skin or wind a soft woven bandage round the splint, which will effectually prevent the irritation often caused by this material.

Any complaint of pain on the part of a patient recently put into splints must be promptly attended to. Frequently bandages do not appear to be too tight when first applied, or the patient may be too nervous to suggest such a thing, and considerable pain is experienced a few hours afterwards. Blueness or swelling of the limb beyond the splint, of course, indicates that the circulation of the blood is being interfered with, and this condition must be quickly relieved. I have seen even a "plaster leg" have to be entirely reapplied very soon after the first fixation, owing to swelling of the limb below the plaster. This condition is liable to occur after the application of any kind of bandage, and it becomes the duty of the nurse to frequently inspect these cases and report any unusual appearance of the skin. In the case of Liston or box splints, the upper portion of the splint should be well padded, and the axillary region dusted with boracic powder, so that no discomfort may be felt by the patient, while he must perforce lie flat upon his back. A great deal of unnecessary suffering often arises from the pressure of the heel upon the splint, in the case of fractures of the tibia or fibula, or when through any disease in the knees, the leg has to be kept at rest for a length of time; this may be avoided by placing a firm pad just above the heel, about as high as the ankle-bone. I have known a strong man kept awake for hours through neglect of this simple precaution.

A Thomas's splint needs to be very carefully applied, so that it may give support to the patient just where it is needed. The bandages often get loose and need tightening, and not seldom the wash-leather itself wears off, especially if the case is a long-standing one.

With regard to extension cases, we need to bear in mind that strapping irritates the skin and also it is only removed with more or less pain, and the traces it leaves generally have to be removed by the aid of turpentine. When applying extension to a limb, I have generally found it a good plan to wind spirally a soft woven bandage round the limb before applying the strapping; this method entirely prevents any irritation, and does away with the soreness so often visible when the strapping is removed. I need hardly say that the ankle bone must be left exposed; I mean, of course, that the bandaging must commence above the ankle, and a bit of wool may be placed over the prominence to avoid any possibility of injury where the skin comes in contact with the strapping. Where extension is applied for the treatment of hip-disease, the patient will be peculiarly susceptible to the slightest jar, or sudden jerk, and in these cases great care must be taken to gently lift the weight while sheets are being changed or when the patient has to be lifted for any purpose whatever. I have known extension made by means of two pieces of strapping joined together on the stirrup. This unfortunate mistake had disastrous results, for the whole affair came to grief during the night. The weight landed on the floor and the next morning a new and better arrangement for extension was made. Moral! never try to make extension with a join in the middle of the strapping.

In the case of children great vigilance is necessary where splints are applied, as so often they are unable to make any complaint of pain, and it is left for the nurse to discover any mischief that may be going on.

During a lengthened experience in children's surgical wards, I learnt the value of box-splints. The child lying as in a frame, can be so easily lifted bodily from the bed, and just as easily turned over on its side while all crumbs are removed, and the back attended to and the bed made comfortable. The house surgeon also pronounced emphatically his approval of these splints, and the nurses' labours were considerably lightened by their use. A piece of lint about a yard and a quarter long, doubled lengthways, makes a most excellent binder for the chest, and if fastened firmly on to the side of the box-splint keeps it in place. Lest the term box-splint may be not familiar to all nurses, I may add that it is merely two long wooden splints, like Liston's, with the pointed ends cut off, and a straight piece at one end joining the two long pieces together, like a picture frame with one end taken out—very simple but most practical.

### Nursing in Small Hospitals.

INTERVIEW WITH THE MATRON OF WEST CORNWALL INFIRMARY. BY OUR COMMISSIONER.

About a quarter of a mile from the market place of Penzance, on elevated ground reached by a continuous gentle ascent, stands the West Cornwall Dispensary and Infirmary. There I sought the matron one bright sunshiny morning. The word "sought" is used advisedly, for the builders had taken possession more or less of the whole place, and it was only by asking directions from men on ladders, and by dodging about under scaffolds, that I succeeded in making my way into the matron's room.

### The New Hospital.

My first remark was naturally as to the building operations, and Miss Whittaker expressed regret that I should have found the hospital in such a disturbed condition. "We started operations," she said, "twelve months ago, but the excavations have been so extensive that there is still a good deal to be done, and we can hardly hope to be quite in order much before next summer."

"I gather that you are rebuilding rather than only improving the hospital?"

"That is so. We are building an entirely new institution, which will be quite up to date in every detail. The Committee were not able to get another suitable site in Penzance, so they bought part of an adjoining field on which, by pulling down a large proportion of the old building, they have been able to build two-thirds of the new, into which we hope to move about Christmas. The remaining part of the Hospital and the Dispensary (which is a large one) will be erected on the ground upon which the hospital now stands."

"How many patients can you receive?"

"At present the hospital accommodates only 19 adults and three children; when the new hospital is complete there will be room for 28 beds and three cots. In addition to a large men's and a large women's ward we shall have two small private wards, both male and female. These will be used for any special case, for a patient requiring extra quiet, or for a paying patient.

### The Class of Patients.

"Do you get many paying patients?"

"A fair number, but they are always accident or operation cases. However, considering that this is only a little hospital, we have patients of a very varied class. Labourers, mechanics, small tradesmen, shipwrecked mariners, or sailors injured on board in discharge of their duty and a few mining accidents. But not so many of these as we

### NURSING IN SMALL HOSPITALS—*Continued.*

used to have, because all the mines except one have now ceased working in this particular district, which includes from Land's End to Helston. At one time we had some typhoid cases regularly every year when the mackerel fishing was on, attributable probably to the water-tanks on board the ships not being kept clean. But now, owing to more knowledge and greater care, we have not had a case sent us from the mackerel smacks for the past three years."

"Have you been here long?"

"Fifteen years. I was trained at Bradford Royal Infirmary. The three years' training was then already in force, and I remained charge nurse for a year and eight months after I had qualified. Afterwards I went to Clayton Hospital, Wakefield, next to the Essex and Colchester Hospital as sister, and for the six years previous to my coming here I was matron at the Male Accident Hospital, Swindon, established by the Great Western Railway Company for those of their employés who need surgical treatment."

"Three years' training is in vogue here now I notice. Was it so when you came?"

### Training in Small Hospitals.

"No, but we soon found the advisability of it. Of course our training is quite different from that given at the large hospitals, and I am always very careful to impress upon prospective probationers that the certificate they will get at the end of their term here will not be equivalent in the eyes of the world to that obtained at a large hospital. But for those who do not wish for the rush and excitement which are characteristic of a large hospital, or who think it probable that they will afterwards go in either for a small hospital or for district or private work, the training they can obtain here will stand them in good stead. This is especially the case with regard to private work. Having so comparatively few patients we are more or less like a happy family, and we are able to dispense with hard-and-fast rules, and study the wants—and I had almost said "fancies"—of those who are sick in a way which is highly desirable in a private house but quite impossible in a large hospital."

"A further point worth mentioning," continued the matron, "is that, owing to the work being of a less arduous character than in an institution of 400 or 500 beds, and less responsibility attaching to a probationer, candidates can begin their training earlier. I work with the nurses every day, so that I am able to see at once if the health of the probationer is suffering from the strain, and also how soon she is capable of doing more important work. Then, with a difficult dressing, I let her see me do it till I think that she can do it herself; then she works whilst I watch her. In this way I am able to find out the weak places in each girl's powers, and I do my best to strengthen them."

"How many nurses are there in this hospital?"

"Three, and we shall need a fourth at least when the new hospital is open."

"On what conditions do you receive them?"

### The Probationers at Penzance.

"They must have had a good education, be thoroughly healthy, and at least 20 years of age. I do not care for them to enter earlier than that, although one of the most competent nurses I ever had—now working in one of the best private homes in London—was only 17 when she came to me. But that was an exceptional case. For the first six months our probationers have no salary and find their own uniform. After that, for the remaining six months they are paid at the rate of £12 a year, but continue to wear their own uniform. The second year their uniform is given them and £14 a year; the third year £18 and uniform. As soon as they are in receipt of a sufficient salary I always urge them to join the Royal National Pension Fund for Nurses, of which I have been a member for some years. I think that it is most essential to impress thrift upon nurses."

"Your head nurse I suppose you get from outside?"

"No, I train all my nurses myself now. When first I came I thought otherwise, but I found the scheme unsatisfactory. Really good nurses thought it hardly worth their while to come to so small a hospital, and I did not care about the second best. So now I train my probationers till they can, if needful, do my work. The only disadvantage is to myself, that I am tied in minor points. The nurses have lectures from the visiting physician, for whom they write examination papers, and if these and their practical work are satisfactory at the end of the three years they are entitled to a certificate; but it is not given to them until they leave the hospital, because then I can say on it how long the nurse has been here. One of my present nurses trained here is in her fifth year. Another probationer after some years here is now sister in a hospital of 600 beds in the North of England."

### Hours of Duty.

"What are the hours of duty?"

"Day nurses come into the wards at 7 a.m. till 8.30 p.m. Besides time for meals, they are off duty one afternoon in each week from two till 9.30, and one afternoon from two to four; also every other Sunday morning, the alternate Sunday being free from five to 9.30. They are allowed out of the infirmary at all hours, when they are off duty, except of course at night. As a matter of fact, they also get a good deal of odd time, for when we have not many patients in I often give them extra leave. The night nurse—there is only one required, and she calls me if anything serious arises—is on duty from 9 p.m. to 9 a.m. She is at liberty to go out of the infirmary every day till 12.30. No probationer is put on night duty till she has thoroughly grown accustomed to her day duties, and the night nurse is changed every month. Before she resumes day work she is off duty from 9 a.m. one day till 7 a.m. the next day."

### What the New Building will be Like.

"And now perhaps you would like to see how far we have progressed in our building," and the matron led the way over into the portion of the new hospital which is nearly completed. Here I noted the big airy wards with African teak floor and tessellated balcony at the end upon which those patients still confined to their beds can be wheeled out and so enjoy the benefit of the beautiful air. The garden is evidently to be a great feature, for nearly all the rooms have steps leading out to it, including the matron's room and the nurses' dining-room. At present the nurses have given up their sitting-room to be used as a temporary operating theatre, for the old theatre has been demolished and the new one is not yet fit for use. The nurses will have large, airy, separate bedrooms under the new scheme, though even at present cubicles are unknown at Penzance, the importance of separate bedroom accommodation for nurses having been always thoroughly recognised by the authorities. Moreover, with wise forethought, provision has been made for the time when yet further developments are required in the hospital, for the roof over the wards is so built that when a second floor needs to be added—at present they are only one storey high—it can be removed and put on again. The administrative block, however, is two storeys high.

### Invalid Cookery and Housekeeping.

As we passed into the kitchen the matron told me that she was glad, towards the end of their time, for her probationers to learn invalid cookery and housekeeping, both of which she thinks it imperative that a nurse should know. Last of all we reached the laundry, all of which is new and up to date, a fact upon which I commented.

"Yes," said the matron, "all appliances needed to wash well and easily are here. This I know from experience."

### Incidents in a Nurse's Life.

**Contributions for this column are invited.**

IN THE NIGHT-WATCHES.

2.30 A.M.—Temperatures, feeds, poultices, duly taken, given, or applied, the one really critical case in the ward sleeping, the mischievous ones—*i.e.* the three small boys—safely snoring, boys and mischief being synonymous, and the snoring stage the only time they are ever quiet, though it sounds paradoxical—my thoughts turned to tea and the kitchen table. Tea for obvious reasons and the table because the ward kitchen boasts no chair. The big square bread-tin with a cloak thrown over the top is too much of a cosy corner to be safe, so is reserved for night sister, who is never guilty of any feeling but the right one on duty.

Well, to resume. Having looked round, made tea, seated myself on the said table with feet well off the ground, the night bell needs must ring. Whir-r-r. How it cut through the silence!—the side-entrance bell too! What did anyone mean by coming round there in the dead of night? But while these thoughts occurred to me, I lighted "Venus" and was half-way down "The Cloister," a long stone corridor leading to the side entrance, for no nurse who values her reputation lets that bell ring twice.

On opening the door a gust of wind and rain beat in my face; but a figure was just discernible in the prevailing blackness, so without more ado I pulled it in and shut the door.

"Now," I said, intending to be stern, "what do you——," but having by this time turned the light on my visitor's face, I stopped amazed. There stood a little pathetic figure, with a face just after the heart of the old masters. From beneath a halo of dark glossy hair looked out two glowing black eyes, in which misery and fear were plainly visible. The childish lips were tremulous, but moved as if to say something. To complete the unexpectedness of the whole, the visitor was clad in the bizarre and, in this case, picturesque garb of an Italian organ-grinder. The head of a Madonna on the shoulders of a little organ-grinder, I silently commented, as I turned to readjust Venus while she recovered her composure. Then—

"It is not usual to call at this hour unless you are very ill," I said, "but can I do anything for you?"

"Baby Lucia—is she better, please? I have no sleep away, and the weather frighten me more," she faltered in very broken English.

"You mean your sister, Baby Consuelo? She is at any rate no worse, and is sleeping quietly. I had just left her when you rang." Her eyes looked up at me in pained reproach.

"My sister, no—my baby, my little one;" and here the poor little mother relapsed into a veritable wail of Italian, of which her "bambino amatissimo" and "paradiso" were chiefly concerned.

"Don't cry," I said; "dry those tears, and we will go and ask Sister if you may see how pretty your bambino looks asleep. Come, follow me, but no noise, please."

We started down the cloister, but presently, thinking I was going too fast, I turned to wait, and found her peering anxiously on either side, vigorously crossing herself.

Seeing me she whispered, "Have you no fear to come down here alone—angry to-night; it is terrible, all dark, the spirits are—Santa Maria!" and she shivered expressively.

"No," I answered, "I have Venus you see," and smiled as I swung her, thinking of those wise words, "Whoso can look on death starts at no shadows." Then, as an afterthought—

"But surely the roads were very dark as you came along; were not you afraid?"

"Yes, very; but," with a contemptuous look at Venus, "there were lamps, and I ran."

Having now reached the ward and asked my visitor to sit outside, I looked round at the patients, and then went to Sister. Of course at first it was Sister's duty to look serious when she heard my tale.

"An inquirer at this hour? You admitted her through the side entrance? If she had not had the face of a Madonna you would have sent her round to the proper entrance; Nurse, Nurse, what next?"

But Sister's seriousness is tempered with sweetness, and though at times she can make one feel an erring atom, she does not leave one without a spark. For which there is more to be thankful than might appear on the surface.

"Well, yes, Nurse; I will come and speak to her." The result being that Signora Consuelo was allowed to look at her baby. Her face was full of apprehension when I opened the ward door, but when she saw her little one sleeping peacefully amid soft pillows and snowy sheets, a cheery fire not far off, accompanied by Master Billy Jones's inhuman snores (surely sufficient to dispel any spiritual element evil or otherwise), and the soothing sounds proceeding from "Pretty Polly's" cot (though it was only her thumbs she was sucking), and the whole ward warm and cosy, her face lit up indeed.

But only a few minutes did I venture to allow her, fearing lest she would be seized with a natural desire to kiss or croon to her baby, so soon drew her away and left her in an empty sideward drying skirt, shawl, and shoes, while I went in search of something hot. Night Sister, however, had forestalled me and was already crumbling bread into boiling milk. She glanced up as I entered and gave voice to some of her seriousness.

"Ah, now, did you ever see such a bit of a mother? Poor wee thing; we will indulge her a bit, Nurse; let her stay until six. I will speak to matron in the morning."

"Shall I scold her for coming to the private entrance, Sister, or will you?"

"Well! of all the hard-hearted little——" Sister began; then, seeing my smile, which I could not keep entirely down my sleeve, she departed with a shake of her head. When I returned to my visitor sorrow and fear were again at war with her. Pushing aside the proferred bowl of bread and milk she kept asking, in broken sentences, if her baby would live. As a matter of fact there was not much chance, for it was a case of acute pneumonia combined with a poor constitution; so all I could honestly say was, that everything possible was being done, that the doctor was very clever and interested in her baby, and so she must hope for the best. That did not satisfy her; for a few moments she sat pondering, then startled me by saying: "But the magic! have you not magic so we can quickly see?"

For some time I could not think what she meant, but when she explained that it was little and bright jumped up or ran down by which one saw whether the patient would live or die I with some hesitancy produced a thermometer. She joyfully clasped her hands and begged me to try it at once. No amount of explanation or reasoning then or afterwards would rid her of the idea that it was supernatural. Finally I left her to get what sleep and comfort she could while I returned to the ward.

Once later I looked in quietly, the bread and milk stood untouched in the hearth, she had fallen asleep with her head on the arm of the chair, like a tired child worn out with crying. After this she was allowed to come every night at 10 P.M. and stay until 6 A.M. Sometimes she would gleefully show me part of the day's earnings and talk of the pretty things she would buy for her baby when it was well.

But, alas! in spite of all efforts the child grew worse and on the fifth night she lost it. Only once have I seen so sad a death-bed.

And after! Poor little child-mother driven through circumstances to play and hear the gay airs of a street organ all day long. Watching, perchance, the dancing feet of other children, while her own little one lay still and silent. Worst of all, I fear, with a firm conviction that if we had only understood the "magic" better or humoured the saints she need not have lost her. Was it, I wonder, in the night-watches that some one wrote, "We need that other world, if we would escape despair in the world that now is."

### Everybody's Opinion.

**[Correspondence on all subjects is invited, but we cannot in any way be responsible for the opinions expressed by our correspondents. No communication can be entertained if the name and address of the correspondent are not given as a guarantee of good faith, but not necessarily for publication. All correspondents should write on one side of the paper only.]**

### AN ISOLATION HOSPITAL WITHOUT A TRAINED NURSE.

Mr. Charles E. Paget, County Medical Officer of Health under the Northamptonshire County Council, writes from the County Hall, Northampton: In The Hospital *Nursing Section* of September 9th, 1905, are some remarkable statements as to an isolation hospital in "Northamptonshire," and editorial comments on "county administration," which require explanation. I shall be glad to learn where the hospital is situated, as it is news to me that there is one of 80 beds over which the County Council has any control.

[There was no reference to the Northamptonshire County Council in our comments, and we have no doubt that if the hospital in question were under its jurisdiction, the state of affairs referred to would be altered.—The Editor, The Hospital.]

"R. A. L. W." writes: I read with amusement the letter which appeared in The Hospital from "M. C. T.," bearing on the subject of an isolation hospital being without a trained nurse. Does "M. C. T." infer that only trained nurses know how to remove, clean, and replace a tracheotomy tube? Surely not. What great difficulty is there in performing this simple matter? There are scores of hospitals in Great Britain where untrained nurses are employed and I think I can say with safety that 50 per cent. of these untrained nurses can abstract, clean and replace such a tube as "M. C. T." refers to. I, in common with others who read her letter to you, should like to know what salary "M. C. T." received prior to her place being filled by an untrained nurse at £40 per annum. Would your correspondent also say how far she had to go when called up on the several occasions she mentions, and how in the meantime the patient managed to live until her valued assistance was procurable. "M. C. T." I think lays too great a stress on the fact that she is a trained nurse. In all the fever hospitals under the Metropolitan Asylums Board, practically all the staff (untrained) nurses are as capable of attending on, and administering to a serious tracheotomy case as any trained nurse is.

### THE REGISTRATION OF TRAINING SCHOOLS.

"A Trained Nurse" writes: Some time ago mention was made in The Hospital, in reference to "Registration," of an idea to register the training schools instead of the nurses. As complete records are kept in most hospitals of those who were trained in them, would not this be a simpler plan than that under consideration? Are there any serious objections or difficulties?

[It is because there are no serious objections to the registration of training schools in the manner described that we supported the principle.—Ed. The Hospital.]

### ONLY A FEVER NURSE.

"Isolation Hospitaler" writes: I have been nursing since 1895, and have had about two years' general training, though not consecutively, as I had to lay up for "nasal polypi." I have done gynæcological and throat work, nursed typhus and small-pox, but my chief experience lies in the direction of diphtheria and typhoid, and I have been charge-nurse of the enteric pavilion of a large hospital for 15 months. I am quite conscious that my training has not been for the regulation period, but I never in any way usurp the post of a three-years'-certificated nurse, nor do I express opinions which would lead people to believe I am a surgical nurse. But ten years' hospital work and experience should make me at least well up in fever work, and I think the tendency of the modern three-years'-trained nurse to look down on any nurse not so trained as necessarily incompetent is much to be deprecated. Others who work in the same sphere as I do must have found it equally galling to hear young women who have only just secured their three-year-certificate remark of nurses with four times their experience, "Oh, she is only a fever nurse!" I feel that I may the more easily write thus because personally I have just been appointed as matron to a good-sized isolation hospital in one of the Home counties, having been successful amongst 50 applicants, which fact may serve as an encouragement to others, now at the bottom of the ladder, who, owing to health or family reasons, are unable to go into a general hospital, turn to fever work, and then find they are considered very inferior folk.

### THE REGISTRATION OF NURSES.

"Certificated Male Nurse" writes: A Scotch certified male nurse, whose letter was published in your issue of September 9th under the above heading, is, I presume from his letter, a mental nurse, as his remarks with regard to what is expected from a competent male nurse shows that he is well acquainted with a "text-book" which is very familiar to all mental nurses who have been preparing for their examination for a nursing certificate. With regard to male nurses and registration, I think that it is now very obvious that to the great majority of them, the position is a perfectly clear one, and whether nurses are going to be registered by the State or not, as far as the matter concerns the certified mental nurses, judging from an extract published from the report of the members of the House of Commons committee on the Registration of Nurses, which appeared in The Hospital of August 12th, it will not affect them in the least. The following is an exact quotation of what appeared in that number of The Hospital:—"The House of Commons Committee have recognised the claims to registration of mental or asylum nurses. They are of opinion that a separate register of 'Registered Asylum Nurses' should be kept by the Nursing Council, to which should be admitted the names of nurses who have served for not less than three years in not more than two asylums, have received a certificate of the Medico-Psychological Association and can produce satisfactory certificates of good character." What does this amount to? It amounts to the fact that the certificate of the Medico-Psychological Association is the mental nurses' "passport" to State registration. The State registered mental nurse will be the exact equivalent of the present medico-psychological registered nurse, and this brings me to another point dealt with by the Scotch nurse. Referring to a certain male nurses' association in Scotland he states, "A good many of these men have never served in either a hospital or an asylum." Now, if this be the case, I venture to answer the question he puts without hesitation, namely, Is it right that doctors should be imposed on by such shams? No! but I think that there is very little chance for the shams described to deceive a gentleman of the status of a doctor, and especially now he has been trained to recognise the genuine article. Doctors will not have counterfeits, and will detect them immediately, and if they wish to satisfy themselves with regard to the male mental nurses they engage it is the easiest thing in the world for them to do so by asking for their certificates or by consulting the registrar of the Medico-Psychological Association. Now that the genuine nurses have united together and formed themselves into co-operations, they have nothing to fear from the coachman, footman, porter, or gateman. Their day is gone.

### A Book and its Story.

MARY E. MANN'S NEW NOVEL.*

Emily Geldart, the heroine of Mrs. Mann's novel, is the orphan daughter of General Geldart, a distinguished soldier, who in dying had left her his good name only, and a small pension, with which to face the world. Trained in a London hospital she is, when the book opens, filling an unemployed interval by staying in a Bloomsbury boarding-house uncertain as to the best means of getting a permanent engagement. The uncongenial environment is well described and her reasons for accepting the attentions of a man that she meets there are all a part of it. Until now she had not received the overtures of other men who were attracted to her with encouragement.

"She prided herself on her practical good sense . . . and yet she had decided that no consideration but the one that she loved him, and desired for the sake of her love to live beside him, should induce her to become any man's wife. . . . She was thirty years of age . . . good-looking but with no accomplishments to bring her prominently before the notice of men, and with no desire to attract them. When, as had happened, now and again, during the past twelve years, a man had, nevertheless, found her attractive . . . she had known how to assume a manner that such a one, if timid at all or doubtful of himself, found forbidding almost. He had shrunk away from the stare of her wide, clear eyes, from her proud unsmiling face, with a shrugged shoulder and had escaped." The common ground of wider interests had drawn her in the present instance into the society of the man Edward Tyrell, who holds a prominent place in her story. "They had laughed together over the idiosyncrasies of many of the members of the boarding-house. . . . Emily, for his amusement, had fitted histories to the few who exhibited traits of nature to lift them above the commonplace. She had enjoyed the man's society among a set whose intellectual calibre was lower than her own because of the ease with which he talked to her, and his evident desire so to talk; because they alone in the house seemed to meet on the common ground of interests more inspiring than the discussion of draughts from doors, of a little more spirituality than the endless topics of the relative values of boarding-house keepers, of the doings of Royalty, of the translations of bishops." One day Emily Geldart comes across an advertisement for a parish nurse. "As the nurse will be required to be in daily association with the family of the clergyman of the parish, it is desirable that she should be a lady by birth and education." She applies for the post and is appointed to it. There are many chapters devoted to her experiences among the ignorant East country folk, and Mrs. Mann indulges in the usual cheap satire at the expense of the rector and his wife, who belong to types common enough in the writings of prejudiced persons, whose experiences of the clergy, as a class, are either nil or too limited to be of value; but to the initiated they will pass as overdrawn caricatures, which servility and vulgar pretension are the predominant features.

Of more interest to readers of The Hospital will be the description of the country quarters apportioned to the parish nurse at Twemleigh.

"The cottage in which two rooms had been hired was one of the oldest in the village of Twemleigh. Heavily thatched, with tiny lattice windows, of which only a small square in the upper right-hand corner of each was made to open, it stood sideways to the road." She writes her impressions as follows: "Who would not be comfortable in a sitting-room whose brick floor is more than half covered by a drugget of chocolate and yellow (taken up on damp days for fear of injury by muddied feet), whose hard and shining-surface 'sofy' is ornamented by an antimacassar of orange and violet wool; whose round table, ricketty on its one leg, and occupying nearly all the space of the room, has for central ornament a blue-glass lamp on a combed wool mat; whose stove is made attractive for the springtime with snipped-out strips of newspaper? In spite of this elegance the bed-room boasting less ornament, and less carpet is more to my taste; and more to my taste still is the kitchen in which my landlord and my landlady, Mr. and Mrs. Susan Pike, sit. She is so much the better man that they are always called the Susan Pikes. There, for the sake of the air which comes in through the open door—blown straight across acres of brown and green fields without interruption, straight, I like to think, from the sea, only twenty miles away, the Susan Pikes being happily absent, I sometimes sit."

Rural life in the parish of Twemleigh, according to Mrs. Mann, was the reverse of the idea usually associated with it. For instance, "Talk of the peace of the country." I assure you there are no two people living in this rural spot who are not ready to fly at each other's throats. Every poor woman is filled to the brim with envy because her neighbour is possibly getting more out of the gentry than she." In her dealings with the sick poor she found that instead of improved methods for their relief being welcomed gratefully they were received with suspicion and accepted grudgingly. "Being a determined young woman she always had her way, but only by the lavishing of much energy, the exhaustion of her entire stock of endurance, and often on the part of the patient and the patient's friends, there was resentment." In these matters also, she was not supported by the elderly doctor of the district, "who had grown old in practice and was used to the poor and their ways and who acquiesced cheerfully in the ignorance he had not time to attempt to correct. . . . It was his misfortune that Emily did care, and began by waging war against ignorance, dirt, and superstition, and raging inwardly and outwardly against the too complaisant doctor." As an outlet to the circumscribed life at Twemleigh, Emily carried on a correspondence with Edward Tyrell which relieved the suppressed irritation that such a life engendered. The isolation from which she suffered is vividly set forth.

The exile has usually some haven to which his heart is anchored and his thoughts directed, from some window or other for nearly every traveller a beacon light shines; but Emily Geldart since her father's death had had no home. A couple of sisters of her father's wrote to her now and then; their letters were filled with the exploits of their own sons and daughters, exacting from her letters in return. In these bare outlines of her comings and goings were given. Who among those correspondents really wanted to hear what she thought or did, or read?"

To a solitary woman, even of strong mind, as Emily Geldart is described as being, the realisation of loneliness has its dangers. This is brought out in her relations with Tyrell; a man, who, had she been living in the social environment to which she was born, would not have crossed her path, probably, or, if he had, would not have been received on an equal social footing. "She neither admired—to get at the heart of the matter—the man nor his appearance. Yet in a manner satisfying to the craving of a lonely woman he belonged to her, and from the seclusion of country "she wrote him long letters . . . . when she should have been sleeping the sleep of the hard-worked and weary in the little chamber under the thatched roof. There readers must seek her who care to follow her story. "The Parish Nurse" will be interesting to those who know the part of the country described and something of the difficulties of this branch of the nursing profession.

* "The Parish Nurse." By Mary E. Mann. (Methuen 6s.)

### Practical Hints.

We welcome notes on practical points from nurses.

### "A WRINKLE WORTH REMEMBERING." BY A NURSE.

In a recent severe case of enteritis which I had the patient could retain nothing, not even her medicine, much less the milk and lime-water. Evacuations were very frequent, accompanied with excruciating pain and bearing down. The doctor prescribed the usual suppositories to act as an astringent as well as an anodyne, which, as is customary, I inserted with vaseline. The bowel was in such a highly inflamed, irritable state that it rejected the suppository from one to two minutes after being inserted. I kept the patient on her back, slightly elevating the knees by means of a small pillow underneath them, then dipped a suppository in water instead of greasing it, and inserted into the rectum, not allowing the patient to move, and, as she was in a very exhausted, feverish condition, I darkened the room completely. My patient retained this suppository, and for two hours there was no evacuation, in which time she had some sleep, the first for nearly 24 hours. Upon the doctor's next visit he expressed his satisfaction with my patient's improved condition, remarking, "It is a simple wrinkle, but worth remembering!" Thinking that some other nurses may have experienced the same difficulty with regard to the retention of suppositories, I should like them to try the water. In these highly inflammatory cases of the bowels and rectum grease acts too much as a lubricant, assisting instead of suppressing evacuation.

### Appointments.

**No charge is made for announcements under this head, and we are always glad to receive and publish appointments. The information, to insure accuracy, should be sent from the nurses themselves, and we cannot undertake to correct official announcements which may happen to be inaccurate. It is essential that in all cases the school of training should be given.]**

Barnsley Union Infirmary.—Miss Eve Whitehead has been appointed charge nurse for the female wards. She was trained at St. Luke's Hospital, Halifax, and has since been staff nurse at the City Fever Hospital, Lodge Moor, Sheffield.

Colne and Holme Joint Isolation Hospital.—Miss Edith Sime and Miss May Turner have been appointed charge nurses. Miss Sime was trained at Glasgow Royal Infirmary, and has since been charge nurse at Ham Green Fever Hospital, Bristol. Miss Turner was trained at Manchester Royal Infirmary, and has been charge nurse at Swallow Nest Fever Hospital, near Sheffield.

Gravesend Hospital.—Miss Jeannie Rennie has been appointed matron. She was trained at the London Hospital, Whitechapel, where she has since been staff nurse and sister. She has also been matron of the Bolingbroke Hospital, Wandsworth Common, and home sister and night superintendent at Birmingham General Hospital.

Isolation Hospital, Wharfedale.—Mrs. M. Sutton has been appointed matron. She was trained at the General Hospital, Blackburn, and has since been nurse at the North Staffordshire Institute, sister at Monsall Hospital, Manchester, sister and housekeeper at the City Hospital, Liverpool, and housekeeper at the Retreat, York.

Pontypridd and Llantwit Fardre Isolation Hospital.—Miss Annie Wood has been appointed matron. She was trained at the London Hospital, and has since been sister at the Plaistow Fever Hospital.

Royal Victoria Hospital, Portsmouth.— Miss Agnes Bywater has been appointed charge nurse. She was trained at the Royal Southern Hospital, Liverpool, and has since been staff nurse at a private surgical hospital in Birmingham.

### Presentations.

The Queen's Nurse at Merthyr Vale Collieries.—Miss Abraham, Queen's Nurse at Merthyr Vale Collieries, was recently presented, on the occasion of her resignation to take up the post of superintendent of the District Nursing Association at Huddersfield, with a public testimonial, consisting of a silver Queen Anne tea and coffee service. The workmen arranged a farewell concert which was largely attended. Amongst those present was Miss Griffiths, formerly matron of Lambeth Infirmary.

### Novelties for Nurses.

(By Our Shopping Correspondent.)

### NURSES' TESTIMONIALS.

Messrs. Lewis and Co., 86 Mansion House Chambers, Bucklersbury, E.C., undertake to provide typewritten copies of testimonials at a reduction of 33⅓ per cent. less than the usual price of 1½d. per folio. Typewritten testimonials look better and are easier to read than handwriting, and a nurse is less liable to make the mistake of sending original testimonials if she has neat copies in her possession.

### AN EXCELLENT ABDOMINAL BELT.

This belt is called the "Ariston Belt," and it is the invention of Nurse Beatrice Kent. It has many advantages to recommend it for use after all cases of abdominal operation. The material used is a firm jean lined with soft flannel. It is very well fashioned, and easily adaptable to variations of figure by the arrangement of bands, buckles, and buttons. All the parts are detachable, and buckles and buttons are so arranged as to be easily removed when the belt requires washing. The belt is patented; it is sold by Messrs. Maw and Sons, and the price is 13s.

### To Nurses.

**We invite contributions from any of our readers, and shall be glad to pay for "Notes on News from the Nursing World," or for articles describing nursing experiences at home or abroad dealing with any nursing question from an original point of view, according to length. The minimum payment is 5s. Contributions on topical subjects are specially welcome. Notices of appointments, letters, entertainments, presentations, and deaths are not paid for, but we are always glad to receive them. All rejected manuscripts are returned in due course, and all payments for manuscripts used are made as early as possible after the beginning of each quarter.**

### TRAVEL NOTES AND QUERIES.

By our Travel Correspondent.

Florence in November (Ida).—You will not get accommodation in a good hotel at the terms you mention. Try the following pensions:—"Internationale," Benoit, Lung Arno Serristori; Mme. Girard, 1 Via Montebello; Pension Giotto, Piazza Indipendenza; Pension Norchi, 20A Via Nazionale. The cheapest hotel I know of is Hôtel de Bologne, Via S. Antonino: terms beginning at seven lire per day. The Italian money will give you no trouble; it is decimal coinage like the French, only reckoned in lire instead of francs. You will find that you usually have small notes instead of silver money, which is very scarce.

### Notes and Queries.

**REGULATIONS.**

**The Editor is always willing to answer in this column, without any fee, all reasonable questions, as soon as possible.**

**But the following rules must be carefully observed.**

1. **Every communication must be accompanied by the name and address of the writer.**
2. **The question must always bear upon nursing, directly or indirectly.**

**If an answer is required by letter a fee of half-a-crown must be enclosed with the note containing the inquiry.**

*Nurses' Pension.*

(197) Will you kindly tell me if I am eligible for election to the Nurses' Pension Fund? I am a fully-qualified nurse, trained in Queen Charlotte's Hospital and now in my 46th year, and should esteem it a favour if you will let me know as soon as possible all particulars as I am anxious to become a member of such a fund if I find I can be.—*Nurse B.*

If you write to the Secretary of the Royal National Pension Fund for Nurses, 28 Finsbury Pavement, E.C., he will give you full information.

*Electrical Treatment.*

(198) Please advise me as to the best practical book giving the nurse's duties in electrical baths, also high-frequency and other electrical treatment.—*Electric.*

"Medical Electricity and the Light Treatment," by Kate Neale, sister-in-charge of light treatment at Guy's Hospital. Post free 2s. 10d. Scientific Press, 28 & 29 Southampton Street, Strand, W.C.

*Maternity Nurse.*

(199) I am a trained maternity nurse, not a midwife, and sometimes have a good time to wait in between cases while I have to refuse many as they all come at once. I should be much obliged if you could give me particulars of the Nurses' Co-operation, or suggest some way of having more regular cases. Do you think it advisable to train as a midwife? I trained at —— about three years ago for maternity. Would it be better to go back there or is there anywhere else that would be better for the Central Midwives Board?—*G. S. P.*

For particulars of the Nurses' Co-operation, write to the Lady Superintendent, 8 New Cavendish Street, W., but to become a member a nurse must have had general training. For further training for the Central Midwives Board examination we should advise you to write for advice to the Secretary, 6 Suffolk Street, Pall Mall, S.W.

*Children's Hospital.*

(200) Can you tell me if I can go into a children's hospital as probationer for 12 months. If so will you please give me an address. I am 20 years of age.—*F. M. H.*

Generally only as a paying probationer. But write to the Matron, Paddington Green Children's Hospital, or for further particulars consult "How to Become a Nurse. The Nursing Profession: How and Where to Train," published by the Scientific Press, 28 & 29 Southampton Street, Strand, W.C.

*Health.*

(201) I am very anxious to become a nurse, but I am sorry to say that four years ago I underwent an operation for peritonitis. I had had no previous illness, and am now strong and enjoying good health. Can you tell me if I would be accepted as probationer in a year or two's time?—*T. M. G.*

Probably, if you could obtain a medical man's certificate that you have good health.

*Nursing Homes.*

(202) Will you kindly let me know name and address of the ladies who received wounded officers into their home in town after the war. Also if there is any good nursing home where a nurse (gentlewoman) in delicate health could help in the work for her board for a few weeks, not Brighton?—*E. C. P.*

This institution is now King Edward VII.'s Hospital for Officers, 9 Grosvenor Gardens, S.W. With regard to your second question you might perhaps find what you want through advertising.

**Handbooks for Nurses.**

| | Post Free. |
|---|---|
| "How to Become a Nurse: How and Where to Train." | 2s. 4d. |
| "Nursing: its Theory and Practice." (Lewis.) ... .... | 3s. 6d. |
| "Nurses' Pronouncing Dictionary of Medical Terms." ... | 2s. 0d. |
| "Complete Handbook of Midwifery." (Watson.) ... | 6s. 4d. |
| "Preparation for Operation in Private Houses." ... | 0s. 6d. |

**Of all booksellers or of the Scientific Press, Limited, 28 & 29 Southampton Street, Strand, London, W.C.**

### For Reading to the Sick.

DAY BY DAY.

God lays a little on us every day;
And never, I believe, on all the way
Will burdens bear so deep,
Or pathways lie so threatening and so steep,
But we can go, if by God's power
We only bear the burden of the hour.

*Anon.*

If we allow ourselves to borrow anxiety from to-morrow we shall find that we have a greater load than we can carry. There is just enough for our full measure of strength in the duty and the responsibility of the one day. If, then, we add to this the burden also of to-morrow, our strength will fail. We do great wrong to ourselves, therefore, when we go out of to-day to get burdens which do not belong to it. Not only are the days short, so that we can go on to eventide with our work or our burden, but they are separated as by an impassable wall, so that there may be no overflowing of one day's care or responsibility into the field of another. If we learn well this lesson of living just one day at a time without anxiety for either yesterday or to-morrow, we shall have found one of the greatest secrets of Christian peace. That is the way God teaches us to live. That is the lesson both of the Bible and of nature. If we learn it it will cure us of all anxiety, it will save us from all feverish haste, it will enable us to live sweetly in any experience.

*J. R. Miller, D.D.*

Night, which seems to us a waste of precious hours, is a time of God's working in us. He draws the veil of darkness that none may see Him when He visits us in loving ministry. He folds us in the unconsciousness of sleep that we ourselves may not know when He comes or how He gives to us the marvellous blessings. Night throws its heavy veil over the lovely things of this world, hiding them from our view. Yet its deep shadow is no stain on the splendour of the day. It is no thief of time, no waster of golden hours, no obscurer of beauty. It reveals as much loveliness as it hides, for no sooner is the sun set, leaving earth's splendour of landscape, garden and forest swallowed up in gloom, than there bursts upon our vision the other splendour of the sky filled with glorious stars. A noble sonnet by Blanco White describes the experience of our first parent as he watched the sinking of the sun to his setting at the close of the first day.

Did he not tremble for this lovely frame—
This glorious canopy of light and blue?
Yet, 'neath a curtain of translucent dew,
Bathed in the rays of the great setting flame,
Hesperus, with the host of heaven, came,
And lo! creation widened in man's view.
Who could have thought such darkness lay concealed
Within thy beams, O sun! or who could find,
Whilst fly and leaf and insect stood revealed,
That to such countless orbs thou mad'st us blind!

## Figures and Tables

**Figure f1:**
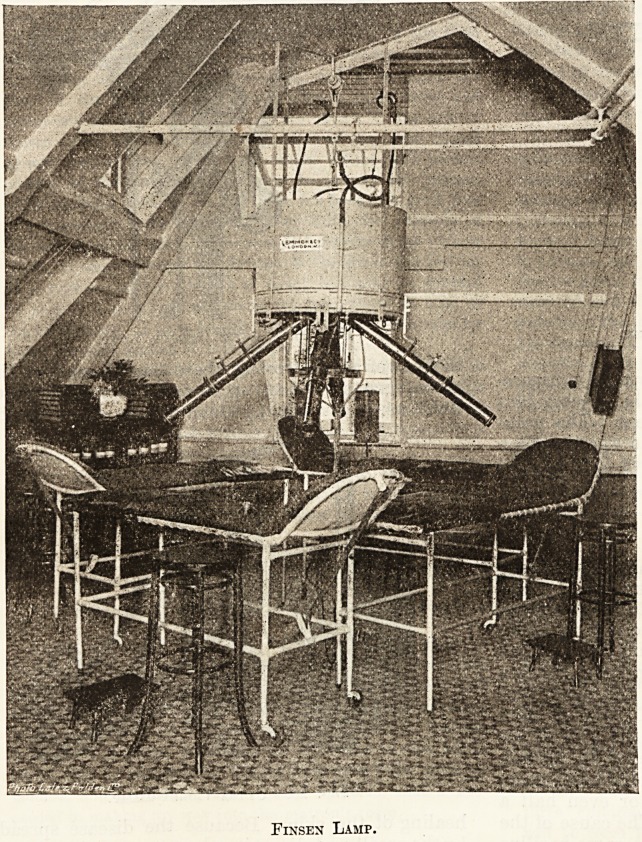


**Fig. 17. f2:**
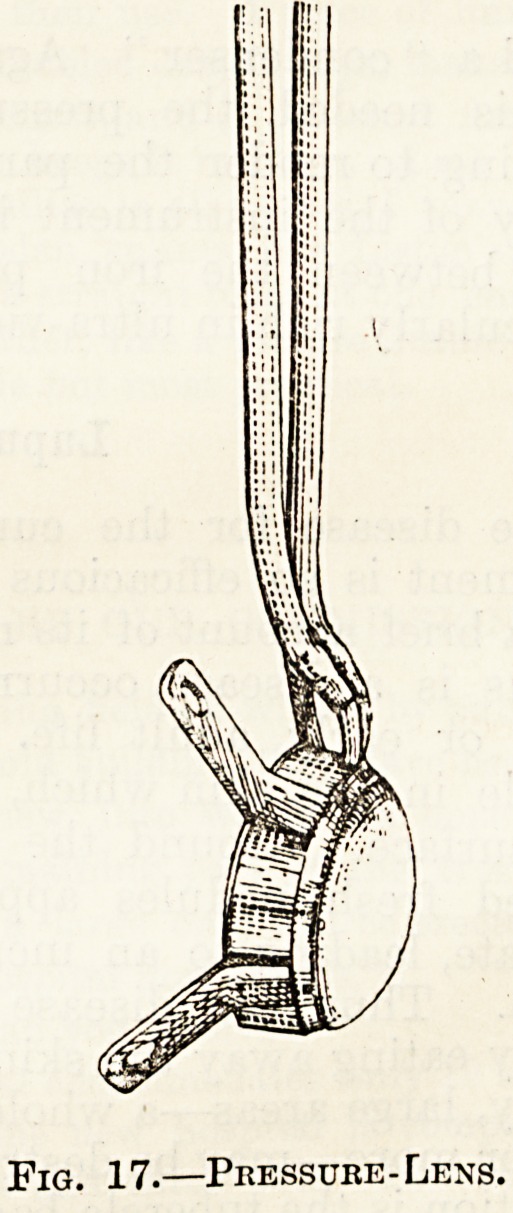


**Fig. 18. f3:**
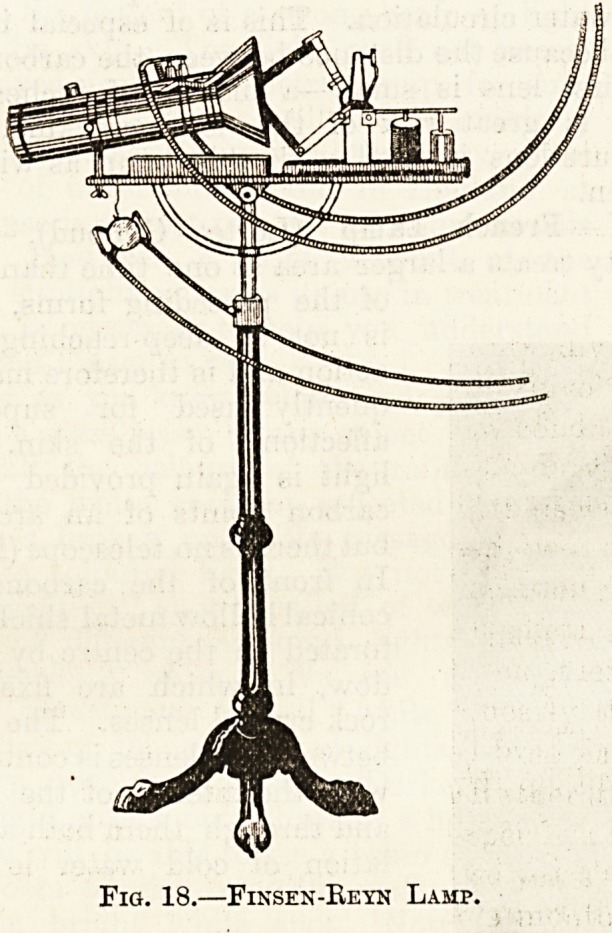


**Fig. 19. f4:**
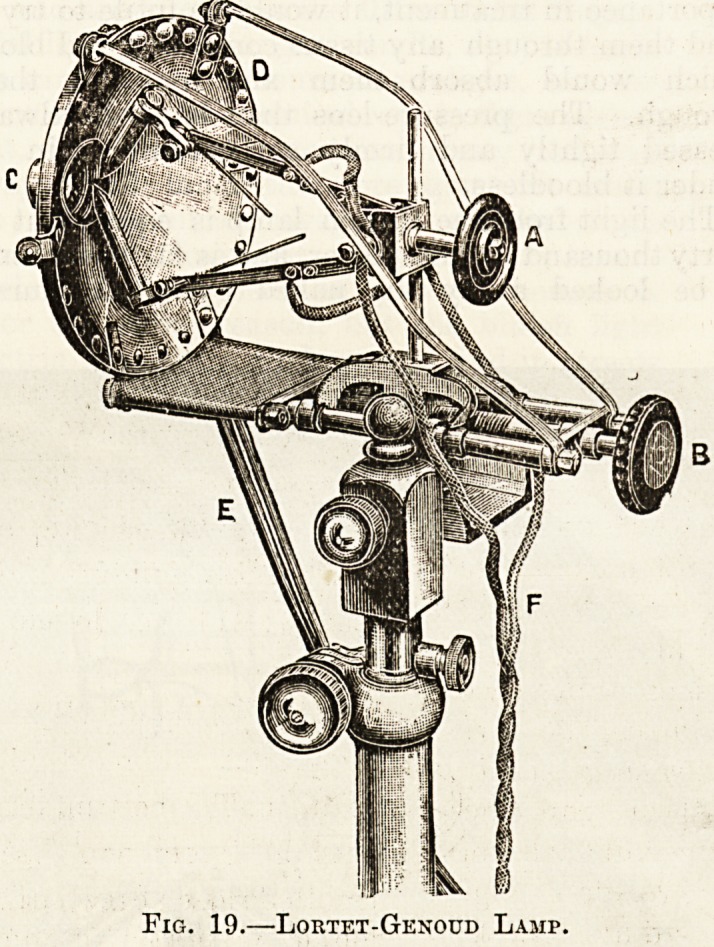


**Fig. 20. f5:**